# Adverse Events Following mRNA COVID-19 Vaccine in 2021 and 2022: A Retrospective Analysis in Costa Rica and Italy

**DOI:** 10.7759/cureus.47834

**Published:** 2023-10-27

**Authors:** Bruno Serrano-Arias, Francesco Ferrara, Esteban Zavaleta, Andrea Zovi, Adolfo Ortiz-Barboza, Roberta Pasquinucci, Sebastián Arguedas-Chacón, Eduardo Nava, Roberto Langella, Giuseppe Russo

**Affiliations:** 1 Research Department, Hospital Clinica Biblica, San Jose, CRI; 2 Pharmacy Department, ASL (Azienda Sanitaria Locale) Napoli 3 Sud, Napoli, ITA; 3 Hygiene Department, Ministry of Health, Rome, ITA; 4 School of Public Health, Universidad de Costa Rica, San Pedro, CRI; 5 Pharmacy Department, ASL (Azienda Sanitaria Locale) Napoli 3 Sud, Nola, ITA; 6 Italian Society of Hospital Pharmacy, Secretariat of the Lombardy Region, Milan, ITA; 7 Strategic Management Department, ASL (Azienda Sanitaria Locale) Napoli 3 Sud, Naples, ITA

**Keywords:** public health and safety, public health, pharmacovigilance, adverse events, pharmacoepidemiology, covid-19 vaccines

## Abstract

Introduction

Pharmacovigilance plays a crucial role in evaluating and monitoring the safety of medicines, which is essential for preventing harm to patients and improving public health. This study aims to compare the pharmacovigilance systems of Costa Rica and Italy and assess the safety profile of coronavirus disease 2019 (COVID-19) vaccines in both countries.

Methods

Data were collected from the official pharmacovigilance platforms in Costa Rica and Italy. Adverse events following immunization (AEFIs) were categorized by system organ class. Reports of suspected AEFIs associated with COVID-19 vaccines were analyzed for the period from January 1, 2021, to December 31, 2022.

Results

Both countries achieved high vaccination rates, with 84.9% in Italy and 92.9% in Costa Rica. A higher proportion of AEFIs occurred in females in both countries, with 53% and 65% in Naples and Costa Rica, respectively. Most AEFIs were observed in individuals aged 18-64 years. The rate of serious adverse reactions was lower in both countries than the international average. However, Naples reported a higher incidence of serious events per 100,000 inhabitants.

Discussion

The study sheds light on the importance of vaccine safety profiling and the significance of a comprehensive understanding of vaccine safety and effectiveness, specific population data, and collaborative strategies to mitigate and improve safety. Additionally, the study highlighted the significance of considering sex and gender when evaluating vaccine safety and efficacy, as sex-specific differences may impact vaccine outcomes.

Conclusion

Continuous pharmacovigilance efforts, collaborative approaches, and comprehensive data analysis are critical in ensuring vaccine safety and efficacy and safeguarding global public health. Lessons learned from the COVID-19 pandemic highlight the importance of proactive measures in addressing emerging challenges in vaccine safety and rollout programs worldwide.

## Introduction

Pharmacovigilance is defined by The World Health Organization (WHO) as the science and activities related to the detection, assessment, understanding, and prevention of adverse effects or any other medicine-related problem [1]. Mechanisms for evaluating and monitoring the safety of medicines in clinical use are critical for preventing or reducing harm to patients and thus improving public health. In practice, this entails having a well-organized pharmacovigilance system in place [1].

After becoming a member of the Organization for Economic Cooperation and Development (OECD), Costa Rica now can compare its healthcare system with that of other countries. There are some health indicators that can be compared between Costa Rica and Italy [2,3]. Both nations have implemented universal healthcare systems that strive to ensure coverage for their entire populations, and they also share comparable rankings in the Human Development Index (HDI). Consequently, this study holds significance not only for providing data on coronavirus disease 2019 (COVID-19) vaccination but also for comparing pharmacovigilance platforms among two healthcare systems having similar setups [2-4].

In Costa Rica, the Ministry of Health regulates pharmacovigilance, while in Italy, the main governmental organization is the Italian Medicines Agency (AIFA). Although different strategies are used to study or learn about adverse drug reactions in Costa Rica and Italy, the most widely used method is the spontaneous reporting of adverse reactions by healthcare providers [5,6]. Adverse drug reactions (ADR), as well as adverse events following immunization (AEFIs) in a vaccinovigilance context and adverse events of special interest (AESIs), are studied by pharmacovigilance for epidemiological reasons and to ensure the safety of treatments. Therefore, since COVID-19 vaccines are a product of global interest, in this analysis, each AEFI is considered an AESI [5,6]. COVID-19 vaccines were brought to market to ensure an effective measure to advance the pandemic, but their safety has been uncertain [7]. For these reasons, examination of databases regarding any AEFIs is critical to monitor their use [7-9].

The pharmacovigilance system in Costa Rica

Costa Rica has a pharmacovigilance regulatory framework circumscribed by the National Pharmacovigilance System (NPS). This structure is governed by two key documents: the Regulation of the National Pharmacovigilance System (No. 35244-S) and the Regulation of Good Pharmacovigilance Practices (No. 39417-S) [5].

Regulation 35244-S details what the NPS is, which is defined as the official pharmacovigilance program in the country that integrates the activities that health institutions conduct to collect or elaborate information on adverse reactions to drugs. For its coordination, it has the NCP of the Ministry of Health [5]. The regulation outlines that information regarding the risks associated with medications can be obtained from various sources, which include the spontaneous notification of individual cases of suspected adverse reactions by health professionals in all health facilities, post-authorization studies of the drugs (including pharmaco-epidemiological studies), and periodic post-marketing safety reports provided by the manufacturer of the product as an update of the safety data in the registration dossier of the medicine. The reporting system in Costa Rica is not limited exclusively to health professionals and pharmaceutical industries [5].

The regulation also provides a detailed explanation of the responsibilities that health professionals must fulfill regarding pharmacovigilance. These responsibilities include reporting any suspicion of adverse reactions that they become aware of during their regular practice and submitting such reports to the NCP using the official channel designated for the notification of suspected ADRs and AEFIs [10]. This directive states that serious adverse reactions should be reported within 24 hours and the remainder within 10 working days, and it is necessary to keep all clinical documentation of suspected ADRs to complete or follow up. Currently, pharmacovigilance reports are made through the Regional Portal for online notification of suspected adverse reactions for human use, called Noti-FACEDRA [5]. Each report is filled with the demographic data of the patient and complete information about the drug, including manufacturer, batch number, dose, reason for use, the date of the event, the type of adverse reaction, the outcome, and the full information of the person responsible for the report [10].

On the other hand, the goal of Regulation 39417-S is to define the bases that contribute to installing a quality assurance system in the activities of the NPS by establishing the obligations and responsibilities that must be fulfilled by the different agents that comprise it to guarantee uniform criteria for the evaluation of notifications and the generation of alerts and to promote the understanding and teaching of pharmacovigilance [10]. The NPS has various responsibilities, which include identifying reported alerts, analyzing those reports, and conducting investigations to determine whether a drug is the cause of an event. If an alert is identified as an imminent public health concern, an investigation and report should be undertaken to take necessary sanitary measures [10]. This regulation also defines that all health service providers must have a pharmacovigilance manager. The person in charge of pharmacovigilance in each health center must, among other things, receive notifications of suspected ADRs/AEFIs, process those ADRs/AEFIs, collect necessary clinical information related to ADRs/AEFIs, and submit reports to the NCP [11].

The new pharmacovigilance system in Italy

The National Pharmacovigilance Network (NPN) is Italy's post-marketing surveillance database for the collection, management, and analysis of reports of suspected ADRs and AEFIs from healthcare professionals and citizens [6]. The new NPN, the web-based portal of the AIFA that allows online reporting of suspected ADRs/AEFIs, has been operational since June 2022 [12].

The new NPN comes into existence by virtue of the process of adapting the old NPN to the international standard ISO Individual Case Safety Report (ICSR) ICH E2B (R3) format, required by Article 26(2)(a) of Implementing Regulation (EU) n. 520/2012 [6]. This E2B(R3) format has been mandatory since June 30, 2022, in all EU countries to send and receive reports of suspected ADRs/AEFIs to and from EudraVigilance (EV), the system for the management and analysis of information on suspected adverse reactions to medicinal products authorized or under study in the European Economic Area (EEA), by which the European Medicines Agency (EMA) manages the system on behalf of the European Union (EU) Medicines Regulatory Network, resulting, therefore, in direct liaison with the NPN [6].

The new NPN features advanced functions for managing and analyzing reports of suspected adverse reactions to ensure an increasingly accurate assessment of the safety profile of medicines. It represents the result of years of intensive teamwork that required the involvement of AIFA's top pharmacovigilance and information technology experts [6]. Among the new features, for each individual facility or pharmaceutical company, in addition to the appointment of the local pharmacovigilance manager (RLFV) or pharmacovigilance manager for the pharmaceutical company, a deputy may also be designated to support the RLFV in the management of adverse reaction reports and/or take their place in case of temporary absence [6,12]. With the development of the new NPN, the figure of the user approvers of the region or autonomous province or pharmaceutical company has been introduced, who have the task of enabling or disabling the use of NPN users belonging to the health structures of their own territorial competence [6,12].

The suspected adverse reaction report form is structured into five main sections: (i) patient information; (ii) information on adverse reactions; (iii) drug information; (iv) description of the case and any additional information; and (v) information on the type of report and the reporter [6,12]. Once the report has been submitted through the new platform online, the RLFV of the facility to which the reporter belongs will not receive any notification via e-mail about the successful entry of a new report, but the report will be present in the "Online Reporting List" of the NPN management panel, waiting to be validated by the RLFV. The new platform of the NPN not only allows a fast sending of reports but also reduces the workload of the RLFV related to the management and insertion of paper reports in the AIFA database [6,12].

To consider a report valid from a regulatory perspective, it must contain at least four minimum elements (minimum validation criteria), which are mandatory under current regulations: (i) a patient (at least one piece of information among initials, sex, date of birth, age, or gestational period); (ii) a suspected drug; (iii) an adverse reaction; and (iv) a referral [6,12]. The new functions introduced in the operation of the NPN will contribute to the important data analysis for the safety profile assessment of drugs and vaccines and the generation of the "signal" more accurately, if any.

The objective of this analysis is to conduct a retrospective study using databases in Costa Rica and Italy to assess the safety profile of COVID-19 vaccines. Comparing adverse effects in different countries can provide a broader understanding of its safety and effectiveness. Additionally, it can help identify patterns, trends, and potential risk factors that may be specific to certain populations or healthcare systems. Additionally, Costa Rica and Italy can develop collaborative strategies to address and mitigate potential risks. Sharing knowledge and best practices can contribute to improving vaccine safety protocols and enhancing pharmacovigilance efforts in both countries.

## Materials and methods

This study followed the Strengthening the Reporting of Observational Studies in Epidemiology (STROBE) guidelines. We used Artificial Intelligence (AI) to facilitate the translation and paraphrasing of the text into a more suitable and comprehensible form of English. This AI-driven approach to translation and paraphrasing enabled more effective dissemination of complex information, making it accessible to a broader audience and facilitating better comprehension of the research findings in the English language. The AI tool used was ChatGPT 3.5 (OpenAI, Inc., San Francisco, California, United States).

Study population

This analysis aimed to assess the safety profile of COVID-19 vaccines administered during the 2021-2022 pandemic period in Costa Rica and Italy, two countries with an overlapping pharmacovigilance system [6-9]. One group encompasses the entire territory of Costa Rica, while the other exclusively concentrates on a city in Italy. Although the analysis retains significant value and relevance, it's crucial to acknowledge that comparisons aren't carried out under identical conditions with respect to the studied populations. Patient demographic characteristics such as age and sex were considered to stratify the AEFI reports. Data on suspected AEFIs were collected and analyzed from pharmacovigilance platforms and databases in Costa Rica and Italy, covering the period from January 1, 2021, to December 31, 2022. 

Data sources

Data from Italy were sourced from the vaccines Comirnaty, Vaxzevria, Spikevax, and Jcovden, while the Costa Rican data were derived from the brands Comirnaty, Vaxzevria, and Spikevax [9,12-15].

Crucial aspects for further investigation include the respective numbers of COVID-19 cases and deaths, vaccination rates, the variety of available vaccines, and the specific policies and procedures governing vaccination in each country. This information serves as a foundation for comparative analysis, offering insights into the divergent approaches of Costa Rica and Italy to COVID-19 vaccination and potential opportunities for mutual learning [5].

Data collection

Variables collected for this study from entity reports included the population of each region, the number of vaccinated individuals, the number of vaccine doses administered, and reported adverse reactions. In Costa Rica, data collection was conducted at a national level with an analyzed population sample of approximately 5.2 million inhabitants, encompassing all vaccine doses administered during the two pandemic years, and was provided by the Ministry of Health authorities [13,14]. As a study limitation, in Italy, data collection was specifically conducted at Naples 3 South Local Health Authority (ASL Napoli 3 Sud), where the analyzed population sample consists of approximately one million individuals. The analysis utilized the new NPN, and reports of suspected AEFIs associated with mRNA vaccines were extracted [15].

Reporting systems

In Costa Rica, the Ministry of Health oversees the collection and publication of vaccination data, updated weekly on its website. Conversely, in Italy, the National Institute of Health manages this responsibility, providing daily updates on vaccination data through its website. AEFI descriptions, including diagnosis and symptoms, were coded using the Medical Dictionary for Regulatory Activities (MedDRA) and categorized by system organ class [13]. The severity of AEFIs was assessed based on the WHO criteria [1,13].

Data analysis

The collected variables were entered into Microsoft Excel datasheets (Microsoft Corporation, Redmond, Washington, United States). A descriptive analysis including rates and percentages was performed. In addition, the rates of adverse events reported per 10,000 inhabitants for each population were calculated. This is especially useful for examining experimental data and facilitating comparisons between various data sets in a standardized way.

## Results

Based on the population data presented in Table 1, retrieved from the authorities of both regions in December 2022, it was registered that in Italy, at ASL Napoli 3 Sud, 84.9% of the total population was vaccinated, whereas Costa Rica had achieved a vaccination rate of 92.9% during the same period.

**Table 1:** Overall Reported Vaccination Data in Italy and Costa Rica from 2021 to 2022. *The data were retrieved in December 2022.

	Italy (Naples)	Costa Rica
Total population*	1,044,024	5,212,167
Amount of people vaccinated	886,354 (84.90%)	4,799,835 (92.10%)
Doses administered	2,345,771	12,666,723

In terms of gender, the collected data presented in Table 2 do not exhibit a trend in the distribution of reported adverse effects according to this classification.

**Table 2:** Gender-specific ADR in Italy and Costa Rica from 2021 to 2022. The data were retrieved in December 2022. ADR: adverse drug reactions

	Italy	Costa Rica
Male	721 (46.5%)	4221 (33.98%)
Female	823 (53.1%)	8115 (65.34%)
Not available	6 (0.40%)	84 (0.86%)

The pharmacovigilance report obtained from Italian authorities provided ADR age stratifications: 12-17 years, 18-64 years, and ≥65 years. This result indicated that a significant majority of adverse effects (79.7%) occurred in the population aged 18-64 years. In contrast, the age-specific information obtained from Costa Rica only distinguished between patients below or above 12 years, making it unfeasible to standardize the data for a direct comparison.

Table 3 shows a comparison between both countries in terms of age distributions, displaying differences in the proportion of the aged population to open the discussion and establish a connection with the severity data of adverse effects presented in Table 4. It is worth noting that Costa Rica has reported 2.2 times the number of events. Interestingly, the population of Italy stands out, exhibiting 6.5 times the number of serious events per 10,000 inhabitants.

**Table 3:** Comparison of Age Distribution Between Costa Rica and Italy in Study Period. [14,15]

Age Group (years)	Costa Rica Population, n (%)	Italy Population, n (%)
0-9	662,701 (12.71)	4,455,970 (7.57)
10-19	736,889 (14.14)	5,580,598 (9.48)
20-29	797,586 (15.30)	5,922,403 (10.06)
30-39	848,698 (16.28)	6,567,037 (11.16)
40-49	713,112 (13.68)	8,132,790 (13.81)
50-59	602,135 (11.55)	9,584,425 (16.28)
60-69	474,522 (9.10)	7,873,266 (13.37)
70-79	263,573 (5.06)	6,125,509 (10.41)
80-89	99,975 (1.92)	3,741,059 (6.35)
90+	12,976 (0.25)	887,693 (1.51)
Total	5,212,167 (100)	58,870,750 (100)

**Table 4:** ADR rates per 10,000 Vaccinated Individuals in Italy and Costa Rica in 2021 to 2022. ADR: adverse drug reactions

ADR	Italy	Costa Rica
Total	17.49	25.88
Serious	1.82	0.28
Non serious	15.67	25.60

Table 4 shows the ADR rates per 10,000 vaccinated individuals, and Table 5 displays the reported AEFIs categorized by system organ class in each region during the study period. It is worth noting that when arranging the systems in descending order, differences are observed in the major affected systems, both in terms of incidence ranking and the percentage comparison between countries. Notably, in Costa Rica, 18.16% of events were reported in the category of metabolism and nutrition disorders, contrasting with only 1.23% in Italy.

**Table 5:** Total AEFIs Reported by System Organ Class in Italy and Costa Rica Between 2021 and 2022. AEFI: adverse events following immunization

	Italy					Costa Rica					
System Organ Class	Total, n (%)	COMIRNATY, n (%)	VAXZEVRIA, n (%)	SPIKEVAX, n (%)	JCOVDEN, n (%)		Total n (%)	COMIRNATY, n (%)	VAXZEVRIA, n (%)	SPIKEVAX, n (%)	
General disorders and administration site conditions	326 (21.03)	189 (57.98)	99 (30.37)	25 (7.67)	13 (3.99)		1302 (12.66)	1149 (88.25)	144 (11.06)	9 (0.69)	
Nervous system disorders	172 (11.10)	101 (58.72)	49 (28.49)	21 (12.21)	1 (0.58)		2481 (24.13)	1370 (55.22)	1061 (42.77)	50 (2.02)	
Musculoskeletal and connective tissue disorders	131 (8.45)	99 (75.57)	16 (12.21)	13 (9.92)	3 (2.29)		2023 (19.67)	1206 (59.61)	777 (38.41)	40 (1.98)	
Vascular disorders	121 (7.81)	91 (75.21)	19 (15.7)	9 (7.44)	2 (1.65)		-	-	-	-	
Cardiac disorders	114 (7.35)	94 (82.46)	6 (5.26)	9 (7.89)	5 (4.39)		50 (0.49)	-	45 (90)	5 (10.00)	
Blood and lymphatic system disorders	101 (6.52)	87 (86.14)	8 (7.92)	6 (5.94)	-		289 (2.81)	246 (85.12)	40 (13.84)	3 (1.04)	
Respiratory, thoracic and mediastinal disorders	98 (6.32)	78 (79.59)	13 (13.27)	7 (7.14)	-		463 (4.50)	353 (76.24)	107 (23.11)	3 (0.65)	
Skin and subcutaneous tissue disorders	98 (6.32)	81 (82.65)	12 (12.24)	3 (3.06)	2 (2.04)		621 (6.04)	481 (77.46)	130 (20.93)	10 (1.61)	
Gastrointestinal disorders	92 (5.94)	73 (79.35)	8 (8.7)	11 (11.96)	-		568 (5.52)	365 (64.26)	192 (33.8)	11 (1.94)	
Ear and labyrinth disorders	80 (5.16)	57 (71.25)	18 (22.5)	3 (3.75)	2 (2.50)		619 (6.02)	418 (67.53)	186 (30.05)	15 (2.42)	
Psychiatric disorders	34 (2.19)	18 (52.94)	7 (20.59)	9 (26.47)	-		-	-	-	-	
Infections and infestations	29 (1.87)	22 (75.86)	5 (17.24)	2 (6.90)	-		-	-	-	-	
Investigations	28 (1.81)	11 (39.29)	14 (50.00)	2 (7.14)	1 (3.57)		-	-	-	-	
Reproductive system and breast disorders	27 (1.74)	21 (77.78)	4 (14.81)	2 (7.41)	-		-	-	-	-	
Eye disorders	25 (1.61)	13 (52.00)	8 (32.50)	3 (12.00)	1 (4.00)		-	-	-	-	
Immune system disorders	22 (1.42)	17 (77.27)	3 (13.64)	2 (9.09)	-		-	-	-	-	
Injury, poisoning and procedural complications	20 (1.29)	15 (75.00)	2 (10.00)	1 (5.00)	2 (10.00)		-	-	-	-	
Metabolism and nutrition disorders	19 (1.23)	15 (78.95)	4 (21.05)	-	-		1.867 (18.16)	1.025 (54.90)	812 (43.49)	30 (1.61)	
Renal and urinary disorders	13 (0.84)	12 (92.31)	1 (7.69)	-	-		-	-	-	-	
Total (T)	1.550 (100.00)	-	-	-	-		10.283 (100.00)	-	-	-	

## Discussion

In the realm of public health, the effective prevention and treatment of diseases and conditions often involve the administration of medications to ameliorate symptoms, reduce morbidity and mortality, and shorten hospital stays [16]. Specifically, in the context of vaccines, the importance of monitoring immunized populations to comprehend, detect, assess, and prevent adverse effects is also significant for improving public health and has proven to reduce harm to patients [16,17]. The importance of implementing a robust vaccine surveillance system is widely acknowledged. Nevertheless, the lessons learned from the recent pandemic have brought to light new demands, presenting a challenge to enhance the entire system and ensure safer vaccine utilization for the benefit of patients [17].

The COVID-19 pandemic sparked a global surge in demand for preventive vaccines, making it a top priority for governments, academia, and the pharmaceutical industry. Remarkably, within less than a year of the pandemic's declaration, vaccines received emergency approvals, and vaccination campaigns were swiftly initiated as soon as Phase III safety and efficacy data became available, approximately 10 months after the respective trials commenced [18]. The rapid development and approval of COVID-19 vaccines were facilitated by several factors, including the streamlining of regulatory processes, the redesign of sequential development approaches, insights gleaned from previous pandemics, and prior advancements in novel vaccine platforms [18,19]. Ensuring long-term safety monitoring became a critical focus as outlined by the United States Food and Drug Administration (FDA) and EMA, necessitating pharmacovigilance and post-marketing safety studies, as mandated by regulatory authorities [18]. Hence, comparisons such as those proposed in this study hold significant relevance.

The comparison between these healthcare systems holds significant importance, particularly following Costa Rica's membership in the OECD, which allows for insightful evaluations of its healthcare practices in relation to other countries. Remarkably, Costa Rica and Italy exhibit notable similarities in various health indicators [2,3]. Both nations have implemented universal healthcare systems with the overarching goal of providing comprehensive coverage for their entire populations. Additionally, they share comparable positions in the HDI [2]. Given these similarities, a focus on comparing pharmacovigilance system data becomes highly pertinent.

Analyzing and contrasting the pharmacovigilance practices of these two countries can offer valuable insights into the safety and monitoring of pharmaceutical products. By understanding their approaches to collecting and analyzing drug safety data, opportunities for mutual learning and the enhancement of global pharmacovigilance efforts can be identified. Such a comparative study can facilitate the identification of best practices and potential areas for improvement, contributing to the promotion of safer and more effective healthcare practices in both countries and beyond [2].

Table 1 presents the comprehensive data for both studied regions in 2021 and 2022, providing essential information to calculate the total vaccinated population. When comparing the information provided by the authorities, Italy (84.9%) and Costa Rica (92.9%) show a higher percentage of vaccinated population than the worldwide statistics [20,21]. Data reported in July 2023 indicate that 70.3% of the total global population had received at least one vaccine dose. In contrast, both Italy and Costa Rica have made remarkable strides in their vaccination efforts, surpassing the global average. This noteworthy achievement underscores the dedication and success of these regions' vaccination campaigns in safeguarding their citizens against infectious diseases [20,21].

Upon analyzing the data presented in Table 2, an interesting observation arises: the occurrence of adverse effects is higher among females only in the Italian population of Naples. This observation is consistent with global data, which highlights that up to 68% of patients reporting adverse effects are women [22-26]. Various authors have discussed the importance of considering sex and gender considerations in the design and implementation of studies related to the development and impact of new vaccines for successful global vaccine rollout. Neglecting to acknowledge the significant implications of sex and gender on vaccine efficacy, safety, and implementation can lead to adverse outcomes. Adopting a sex/gender-sensitive approach in these studies can address vaccine hesitancy, improve vaccine coverage, and enhance the effectiveness of vaccination efforts [22-26].

Age-stratified ADR reports were collected; however, a direct comparison of results from different countries was not feasible due to the lack of standardization in reporting. Nevertheless, it is noteworthy that the majority of ADRs were observed in the population aged 18-64 years. This aligns with the initial target population for vaccination campaigns, which prioritized healthcare workers, frontline workers, the elderly (especially those residing in care facilities), individuals with underlying health conditions, and essential workers. These groups were given priority for COVID-19 vaccination due to their increased risk of exposure and vulnerability to the virus [24].

Table 3 presents a comparison of age distribution between Costa Rica and Italy during the study period, offering valuable data for analysis and discussion. When examining the age distribution of both populations, noticeable differences in their age distributions are observed. In Costa Rica, a higher proportion of individuals, approximately 31.58% of the population, fall within the age group of 20-39 years. Conversely, Italy has a larger percentage of individuals, approximately 30%, in the age group of 50-69 years. Costa Rica's age distribution is more balanced across various age groups, without any group significantly dominating. In contrast, Naples (Italy) has a higher proportion of individuals aged 70 years and above in the population, indicating a larger elderly population compared to Costa Rica [14]. In this line, Costa Rica reports a rate of reported adverse effects that is over twice that of Naples per 100,000 inhabitants. In contrast, Naples has 6.5 times more serious events per 100,000 inhabitants. This discrepancy can be attributed to the age disparities between the two populations, knowing that it is well established that younger populations tend to exhibit a higher occurrence of adverse effects [22].

After comparing the data presented in Table 4 with the analysis of serious adverse events reported in the placebo-controlled, phase III randomized clinical trials of mRNA COVID-19 vaccines in adults, it becomes evident that both Italy (1.82 ADR/10,000 inhabitants) and Costa Rica (0.28 ADR/10,000 inhabitants) had a lower rate in this analysis than the internationally reported rate, where the combined mRNA vaccines were associated with a rate of 12.5 serious adverse events per 10,000 vaccinated individuals [22]. These differences could be attributed to the many ways serious adverse effects are classified worldwide. Notably, the CDC defines five types of serious adverse effects related to this vaccine: anaphylaxis, thrombosis with thrombocytopenia syndrome, myocarditis, and pericarditis, and Guillain‒Barré syndrome. It is for this reason that it is necessary to continue with post-marketing studies related to pharmacovigilance on these issues [26].

It is well known that the chances of experiencing a serious adverse reaction, such as one in 100,000, are low, which is why a rate lower than one in 100,000 inhabitants is considered low. To provide context, anaphylaxis following the first dose of the Pfizer COVID-19 vaccine occurred in approximately 1.1 per 100,000 doses administered [27-29]. In this case, it is evident that Naples has a high incidence of serious adverse effects.

Additionally, a study was conducted on a cohort of 19,586 individuals who received the COVID-19 vaccination. The findings indicated that serious adverse effects, such as anaphylaxis or allergies, were infrequent [22]. Notably, adverse effects were more prevalent among those who had received the full vaccination dose and were more commonly observed in participants of younger age, female gender, and with a history of prior COVID-19 [19,22]. These results are consistent with data obtained from randomized clinical trials and government-sponsored surveillance of vaccine safety [22].

Table 5 displays the AEFIs categorized by System Organ Class. They have been arranged in descending order of incidence in the Italy column to facilitate comparison with the data from Costa Rica. The array of adverse effects is comparable to those identified in systematic reviews, meta-analyses of randomized trials, and reporting [22]. Likewise, the most frequent events are concentrated in three categories that coincide across countries: general disorders and administration site conditions, nervous system disorders, and musculoskeletal and connective tissue disorders. These effects are well documented in the literature as the most common conditions following immunization [27].

On the other hand, naturally, vascular events, cardiac disorders, and blood and lymphatic system issues appear after the ones already mentioned in terms of significance. This aligns with the authorities' statements regarding the importance of monitoring these types of events, as they classify them as serious events that pose a risk to patients' lives. Thus, it emphasizes the importance of preventing and mitigating events of this nature in vaccinated populations [26]. Interestingly, Costa Rica stands out with 18.6% of events related to Metabolism and Nutrition Disorders, compared to only 1.23% in the case of Naples. This discrepancy arises from the process of harmonizing the data for presenting these results. As distinct categories exist between countries, the events reported by Costa Ricans as "fever and chills" were included in the Metabolism and Nutrition Disorders category because they are linked to metabolism and not necessarily to musculoskeletal or other conditions [30].

While it is true that the data collected on mortality is a limitation of this study, it sparks a discussion that has generated controversy in recent years. Several studies have reported associations between COVID-19 vaccination and the risk of cardiac diseases; however, the impact on mortality remains unclear [31,32]. International data continue to show that there are no significant increases in mortality rates for the doses of different vaccine manufacturers when combined. In fact, a decrease in incidence has been demonstrated. Similarly, there was a reduced risk of hospital death in the first two weeks after vaccination [31].

## Conclusions

The study sheds light on the importance of vaccine safety profiling and the significance of a comprehensive understanding of vaccine safety and effectiveness, specific population data, and collaborative strategies to mitigate and improve safety. Overall, this manuscript underscores the significance of ongoing pharmacovigilance efforts, collaborative approaches, and comprehensive data analysis in maximizing the safety and efficacy of vaccines, which are crucial for protecting global public health. The lessons learned from the COVID-19 pandemic highlight the necessity for a vigilant and initiative-taking approach in addressing emerging challenges in vaccine safety and promoting successful vaccine rollout programs worldwide.
